# Context-aware KazRoBERTa with transformer-based candidate re-ranking for Kazakh spelling correction

**DOI:** 10.3389/frai.2026.1925050

**Published:** 2026-10-02

**Authors:** Zhanar Azhibekova, Daniyar Sultan, Laura Baitenova

**Affiliations:** 1 Kazakh National Medical University named after S D Asfendiyarov, Almaty, Kazakhstan; 2 Narxoz University, Almaty, Kazakhstan; 3 Turan University, Almaty, Kazakhstan

**Keywords:** candidate re-ranking, KazRoBERTa, low-resource language, masked language transformer, NLP, spelling correction

## Abstract

Automatic spelling correction for Kazakh is difficult because productive morphology, vowel harmony, and Cyrillic keyboard substitutions generate many orthographically plausible alternatives. This study proposes Context-Aware KazRoBERTa, a three-stage framework that combines token-level error detection, linguistically constrained candidate generation, and masked-language-model candidate re-ranking with a shared Transformer encoder. Candidate generation integrates inverse ka2ru mapping, vowel-harmony restoration, Optimal String Alignment distance, and lexicon filtering; contextual probability, orthographic similarity, and lexical frequency are then combined for selection. Evaluation was performed on a synthetic corpus of 2,615,771 parallel sentence pairs derived from 922,798 clean sentences. On the held-out synthetic test set, the framework achieved 97.7% detection F1-score and 98.8% detection accuracy, while sentence correction accuracy reached 85.8%, word correction accuracy reached 95.6%, and character error rate decreased to 1.86%. The 95% Wilson interval for sentence correction accuracy was 85.36–86.23%. Relative to the reported two-stage baseline, the 5.6-point sentence-accuracy margin implies a conservative continuity-corrected McNemar lower bound of χ^2^ > = 230.3 (*p* < 0.001) across all paired contingency tables compatible with the reported rounded margins. Ablation results attribute the largest gain to contextual re-ranking, particularly for deletion and substitution errors. The evidence nevertheless remains limited to one fixed-seed synthetic evaluation: no natural-error test corpus, direct ByT5/mT5/NLLB or sequence-tagging baseline, paired prediction archive, repeated-run variance, or measured latency benchmark was supplied. The web interface therefore demonstrates system integration rather than external accuracy. The scientific contribution is the Kazakh-specific, parameter-sharing hybrid design; claims of real-world generalization or state-of-the-art superiority require external benchmarking and fully archived configurations.

## 1 Introduction

Automatic spelling correction has become an essential component of modern natural language processing (NLP) systems because spelling errors significantly affect downstream applications, including machine translation, information retrieval, question answering, text summarization, and conversational agents (Li et al., 2024). While substantial progress has been achieved for high-resource languages through large-scale Transformer-based language models, low-resource and morphologically rich languages continue to present considerable challenges (Naeem et al., 2024). Kazakh is an agglutinative Turkic language characterized by extensive inflectional morphology, vowel harmony, and a large number of valid word forms generated from a single lexical root (Toktarova et al., 2024). Furthermore, the widespread coexistence of Kazakh and Russian keyboard layouts frequently introduces visually similar Cyrillic substitutions (ka2ru errors), resulting in spelling mistakes that are difficult to identify using conventional dictionary matching or edit-distance algorithms. Existing rule-based spelling correction systems generally fail to capture these linguistic characteristics, whereas purely neural approaches often require extensive annotated corpora that remain unavailable for many low-resource languages (Liu, 2025; Messaoudi and Nessah, 2024).

Recent advances in contextual language modeling have substantially improved grammatical error correction and spelling correction by exploiting semantic information beyond surface-level lexical similarity. Transformer architectures such as BERT and RoBERTa learn contextual representations capable of distinguishing multiple candidate corrections according to sentence meaning rather than orthographic distance alone (Angizeh and Keyvanpour, 2024; Wahyuningsih et al., 2026). Several studies have successfully adapted masked language models for English, Chinese, and multilingual spelling correction, demonstrating superior performance over traditional edit-distance and statistical approaches (Wang J. et al., 2025; Li et al., 2025). Nevertheless, most existing systems either generate corrections directly from a large vocabulary or rely on predefined confusion sets without explicitly combining linguistic candidate generation and contextual semantic reasoning (Kang et al., 2023). Such limitations become particularly pronounced for Kazakh, where morphological complexity and productive word formation frequently produce multiple orthographically similar candidates with nearly identical edit distances. Consequently, selecting the correct replacement requires understanding the contextual semantics of the entire sentence rather than relying solely on lexical similarity.

To address these challenges, this paper proposes Context-Aware KazRoBERTa with Transformer-Based Candidate Re-Ranking for Kazakh spelling correction. The framework separates three decisions: detecting where an error occurs, generating a linguistically credible candidate set, and selecting the candidate that best fits the sentence. A KazRoBERTa token classifier performs detection; inverse ka2ru mapping, vowel-harmony constraints, Optimal String Alignment distance, and lexicon filtering perform candidate generation; and the shared masked language modeling head ranks candidates using contextual probability together with lexical frequency and orthographic similarity. The contribution is not a new self-attention mechanism or pretraining objective. It is the Kazakh-specific integration of linguistic constraints with a parameter-sharing detector-ranker architecture and a component-level evaluation of the hybrid score. Reported improvements are therefore interpreted only against the lexicon heuristic and two-stage KazRoBERTa baselines on the synthetic held-out set, not as evidence of superiority over all current neural correction systems.

## 2 Related works

Automatic spelling correction has been extensively studied for several decades, beginning with dictionary-based and statistical approaches that relied primarily on lexical lookup, confusion matrices, and edit-distance measures (Qu et al., 2025). Classical methods, including minimum edit distance and noisy-channel models, generate candidate corrections according to character similarity and subsequently rank them using word frequency or probabilistic language models (Hwang and Jeong, 2023; Yin Hong et al., 2024). These approaches remain computationally efficient and interpretable; however, they frequently struggle with context-dependent ambiguities and morphologically rich languages, where multiple valid candidates may share identical edit distances. Statistical language models based on n-grams partially alleviate this limitation by incorporating local contextual information, but their fixed-length context restricts their ability to capture long-range semantic dependencies within complex sentences (Dai et al., 2025; Mi et al., 2024). Consequently, traditional spelling correction systems often produce grammatically valid but semantically inappropriate substitutions, particularly in low-resource languages with productive morphology.

The emergence of deep learning and Transformer architectures has fundamentally transformed spelling correction by enabling contextual language understanding through self-attention mechanisms. Bidirectional Encoder Representations from Transformers (BERT) and its variants learn contextual token representations that significantly outperform recurrent neural networks and statistical language models across numerous language understanding tasks (Rushanti et al., 2025). RoBERTa further improves representation quality through optimized pretraining strategies and larger-scale corpora (Zhang and Xu, 2022). Building upon these models, several studies have introduced Transformer-based grammatical and spelling correction systems that formulate correction as sequence labeling, sequence generation, or masked language modeling problems (Sulistyo et al., 2025; Banerjee et al., 2026). These methods demonstrate remarkable improvements in contextual reasoning; nevertheless, many of them generate corrections directly from the entire vocabulary, increasing computational complexity and occasionally producing semantically plausible but orthographically distant replacements. Furthermore, most existing approaches have been developed primarily for English and other high-resource languages, limiting their applicability to morphologically rich and under-resourced languages such as Kazakh (Xu et al., 2024; Qorbani et al., 2025).

Recent research has increasingly emphasized hybrid correction frameworks that integrate linguistic knowledge with neural language models. Candidate generation based on edit distance, morphological rules, pronunciation similarity, or confusion sets is commonly combined with contextual ranking to improve both efficiency and accuracy (Yang, 2024; Wang Z. et al., 2025). Such hybrid approaches reduce the search space while preserving linguistically meaningful candidates for subsequent contextual evaluation. Nevertheless, existing candidate generation techniques are generally designed for alphabetic languages with relatively simple morphology and therefore do not adequately address language-specific phenomena such as vowel harmony, productive suffixation, and visually similar Cyrillic substitutions that frequently occur in Kazakh writing (Ul Qumar et al., 2025). In addition, relatively few studies investigate parameter-sharing strategies that reuse pretrained Transformer encoders for both error detection and contextual candidate selection, despite their potential to reduce inference cost and model complexity (Unanue et al., 2022; Nath et al., 2026).

Kazakh NLP has recently expanded through educational, vision-language, and translation resources (Yelibayeva et al., 2026; Arystanbekov et al., 2026; Shanshin, 2026), but these studies do not provide directly comparable spelling-correction experiments. More directly, Maxutov et al. (2024) introduced kkWikiSpell and showed that contemporary large language models still struggle with Kazakh spelling correction. The present review of accessible literature did not identify a published Kazakh system that jointly combines Transformer-based token detection, inverse ka2ru and vowel-harmony candidate constraints, OSA filtering, and shared-encoder MLM re-ranking. This is a scoped literature-based novelty claim rather than proof of universal firstness. The proposed framework advances system design for Kazakh, while its empirical contribution remains bounded by the available synthetic evaluation.

### 2.1 Research positioning and contribution boundaries

Recent low-resource correction research establishes substantially stronger neural comparison points than dictionary or edit-distance systems alone. Ingólfsdóttir et al. (2023) found that a byte-level ByT5 model trained first on synthetic data and then on curated real errors outperformed subword alternatives for morphologically rich Icelandic. Luhtaru et al. (2024) showed that pretrained multilingual translation models can exceed similarly sized mT5 correction systems. Nasyrova and Sorokin (2025) adapted efficient Transformer sequence tagging to Russian, while Cissé and Sadat (2024) trained a Transformer spell corrector for Wolof from semi-automatically constructed parallel data. Guan and Greene (2024) further demonstrated the relevance of ByT5 and domain-aware synthetic generation for multilingual character-level correction. These paradigms motivate direct future ByT5, mT5/NLLB, and language-adapted sequence-tagging baselines on identical Kazakh splits.

The framework’s distinguishable contribution comprises four linked elements: a shared KazRoBERTa encoder used for both detection and masked-context ranking; a Kazakh-specific candidate generator that models ka2ru substitutions and vowel harmony; a hybrid semantic-orthographic-frequency score; and an error-type analysis showing where contextual ranking helps most. None of these components alone is claimed as a new general Transformer architecture. The scientific hypothesis tested is narrower: within the reported synthetic Kazakh setting, contextual re-ranking of a linguistically constrained candidate set improves correction beyond distance-based selection while retaining a single shared encoder.

## 3 Materials and methods

The proposed Context-Aware KazRoBERTa framework uses a three-stage architecture for automatic Kazakh spelling correction by integrating contextual error detection, linguistically constrained candidate generation, and Transformer-based contextual candidate re-ranking into a unified pipeline. Unlike systems that directly select candidates using edit distance or lexical frequency, the framework separates where an error occurs from how it should be corrected. This organization constrains the search to detected error positions and a filtered candidate set; the submitted evidence does not quantify its computational savings. Figure 1 shows the full workflow from preprocessing and error detection through candidate generation, contextual re-ranking, and sentence reconstruction.

**Figure 1:**
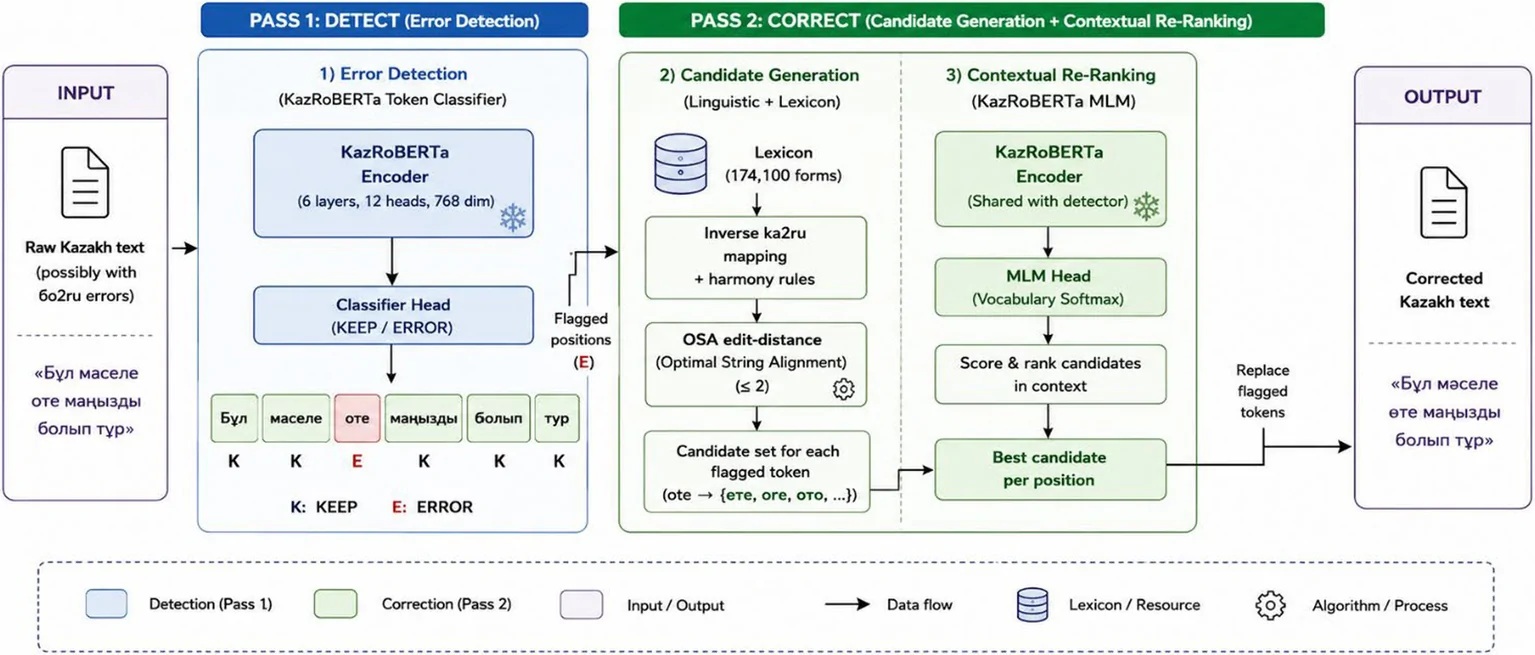
Overall architecture of the proposed context-aware KazRoBERTa spelling correction framework.

Let the input sentence be represented as:


$$X=\left\{x_{1},x_{2},\ldots ,x_{n}\right\},$$

where $x_{i}$ denotes the $i^{th}$ token and n is the sentence length. The objective is to estimate the corrected sentence:


$$\hat{X}=\arg\max P\left(X^{*}|X\right),$$

where $X^{*}$ denotes the latent error-free sentence. Rather than solving this optimization directly, the proposed framework decomposes the correction process into three consecutive subtasks:


$$P\left(X^{*}|X\right)=P\left(D|X\right)P\left(C|D,X\right)P\left(R|C,D,X\right),$$

where D , C , and R denote error detection, candidate generation, and contextual candidate re-ranking, respectively. Such decomposition enables each stage to solve a simpler optimization problem while jointly producing semantically and orthographically correct output.

### 3.1 Synthetic dataset and error modeling

Since large manually annotated Kazakh spelling correction corpora are unavailable, supervised learning requires synthetic generation of parallel sentence pairs. The proposed dataset construction procedure is presented in Figure 2. Initially, a large corpus of clean Kazakh sentences undergoes Unicode normalization, sentence segmentation, duplicate removal, and punctuation normalization. Each cleaned sentence serves as the ground-truth reference from which corrupted samples are generated.

**Figure 2:**
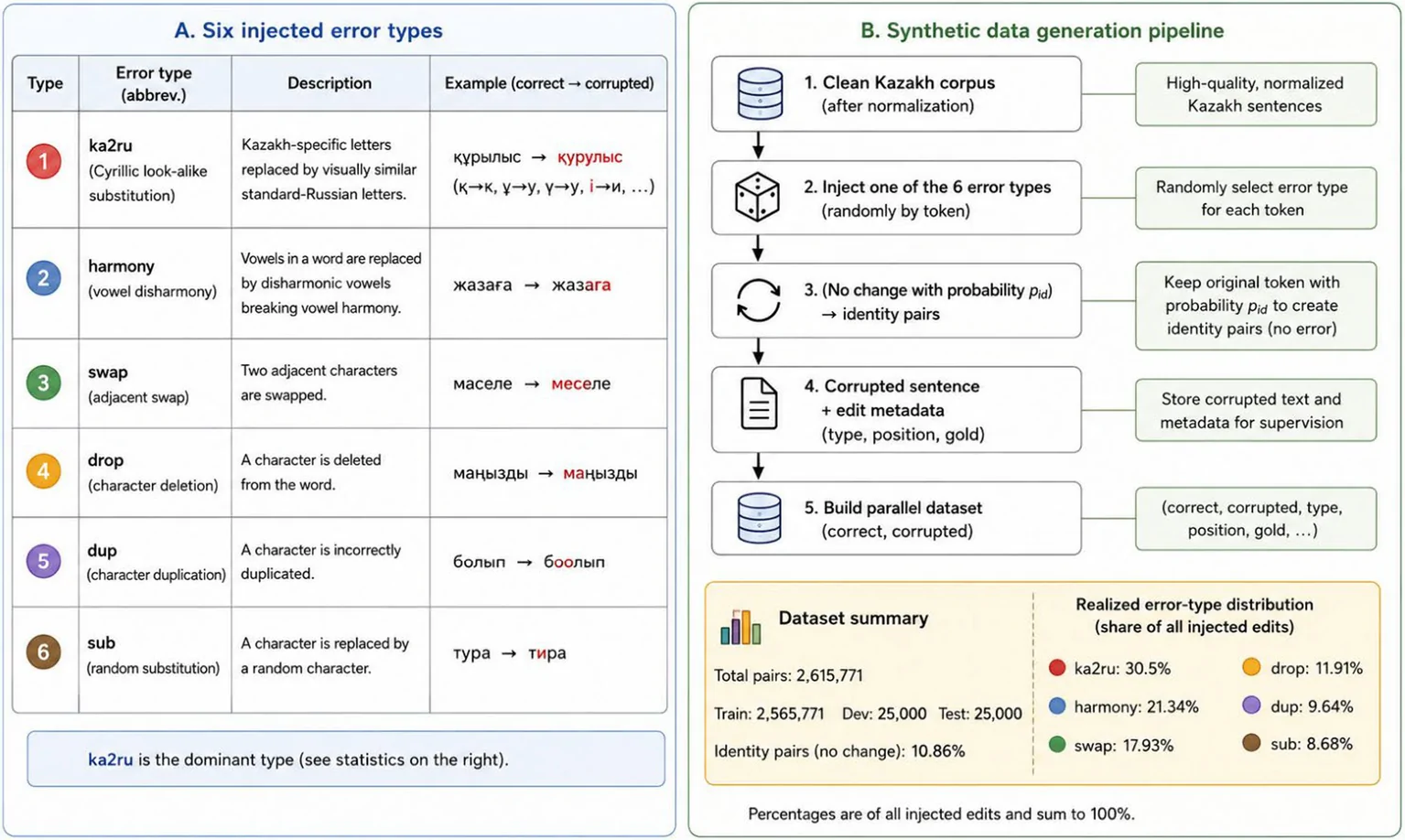
Error taxonomy and synthetic data generation procedure.

Six representative spelling error categories are considered: ka2ru substitutions, vowel harmony violations, adjacent character transpositions, character deletions, character duplications, and random character substitutions. These error types reflect both language-specific and generic spelling mistakes observed in practical Kazakh writing.

For every sentence, characters are corrupted with probability:


$$P_{e}=0.12,$$

While the maximum number of edits is limited to:


$$N_{\max}=3.$$

The corrupted sentence is generated according to:


$$\tilde{X}=F\left(\mathrm{X,}\;\Theta \right),$$

where Θ denotes the sequence of edit operations. Approximately 10.86% of generated pairs remain unchanged:


$$\tilde{X}=X,$$

Preventing excessive correction of already valid words during model training.

The dominant error category corresponds to ka2ru substitutions, which replace Kazakh-specific Cyrillic letters with visually similar Russian characters. The deterministic mapping is defined as:M: {ә, ө, ү, ұ, қ, ғ, ң, і, h} → {а, о, у, у, к, г, н, и, х}

The resulting corpus contains more than 2.6 million aligned sentence pairs divided into training, validation, and testing subsets, providing sufficient diversity for learning contextual spelling correction.

### 3.2 KazRoBERTa-based error detection

The first stage of the proposed framework detects erroneous tokens using a fine-tuned KazRoBERTa encoder. Error detection is formulated as a binary sequence labeling problem where each token receives either a KEEP or ERROR label. Byte-Pair Encoding converts every sentence into subword tokens:


$$T=\left\{t_{1},\;t_{2},\;\ldots ,\;t_{m}\right\}$$

which are embedded as:


$$e_{i}=E\left(t_{i}\right)+P\left(i\right),$$

where E(·) and P(·) denote token and positional embeddings.

The embedded sequence is processed through multiple Transformer encoder layers using scaled dot-product attention:


$$\mathrm{Attention}\left(Q,\;K,\;V\right)=\mathrm{Softmax}\left(\frac{QK^{T}}{\sqrt{d}}\right)V.$$

The resulting contextual representations:


$$H=\left\{h_{1},\;h_{2},\;\ldots ,\;h_{m}\right\}$$

Contain semantic information from the entire sentence. Token classification is then performed using:


$$P\left(y_{i}\right)=\mathrm{Softmax}\left(Wh_{i}+b\right),$$

where W and b denote trainable parameters. The predicted label is


$${\hat{y}}_{i}=\arg\max P\left(y_{i}\right),$$

with


$${\hat{y}}_{i}\in \left\{\mathrm{KEEP,ERROR}\right\}.$$

Training minimizes the binary cross-entropy objective.


$$L_{\det}=\left(-\frac{1}{N}\right)\overset{N}{\underset{i=1}{\sum }}\left[y_{i}\log P_{i}+\left(1-y_{i}\right)\log\left(1-P_{i}\right)\right].$$

Only tokens predicted as ERROR proceed to the correction stage, substantially reducing inference cost.

### 3.3 Linguistically constrained candidate generation

After detecting erroneous tokens, the framework generates correction candidates using the procedure shown in Figure 3. Instead of searching the complete vocabulary, candidate generation exploits language-specific knowledge together with edit-distance matching.

**Figure 3:**
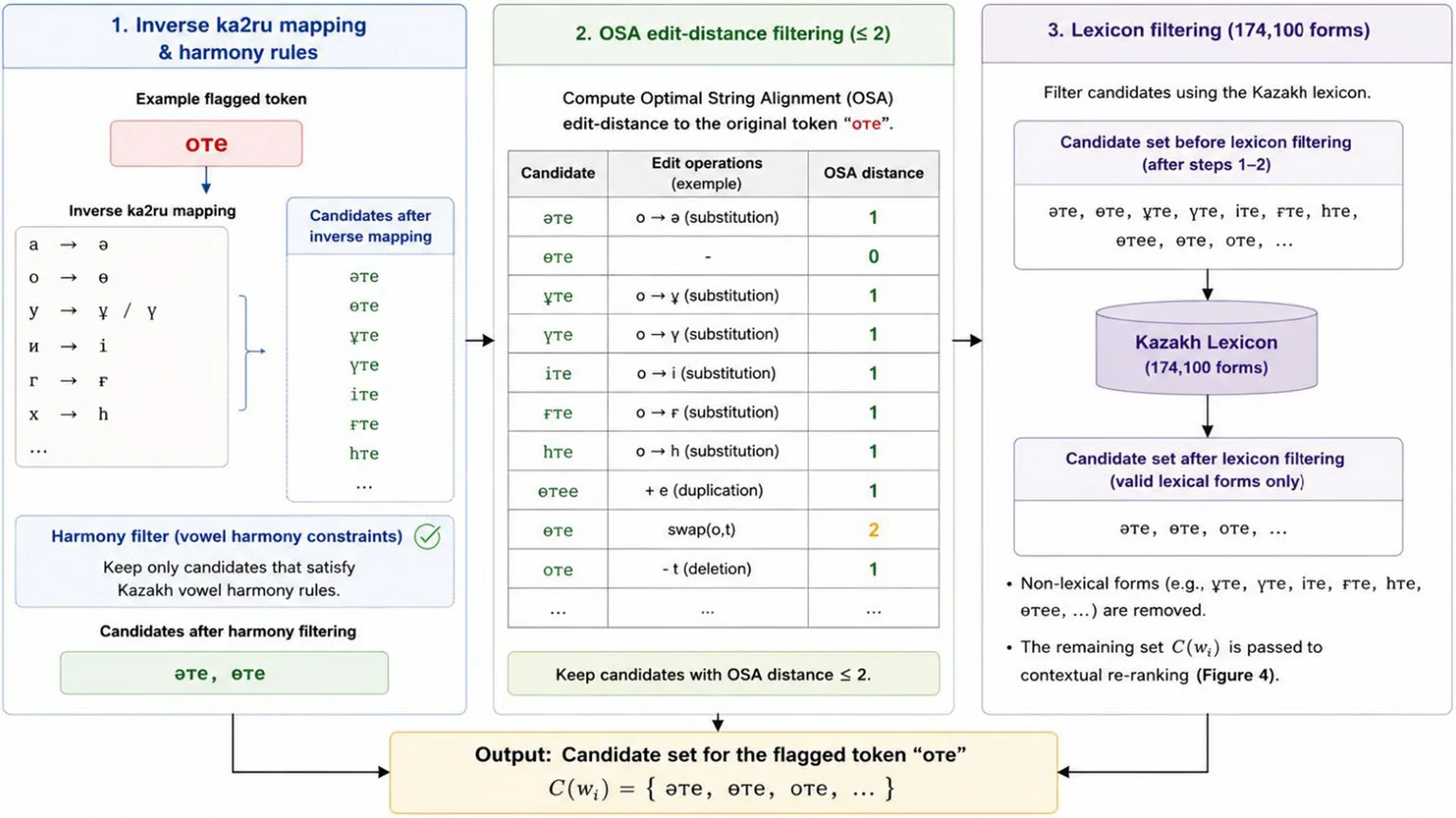
Candidate generation for a flagged token.

For every detected error $x_{i}$ , the candidate set is defined as.


$$C\left(x_{i}\right)=\left\{c_{1},\;c_{2},\;\ldots ,\;c_{k}\right\}$$

Candidate generation combines three complementary strategies,


$$C\left(x_{i}\right)=C_{ka2ru}\cup C_{VH}\cup C_{OSA},$$

where $C_{ka2ru}$ , $C_{VH}$ , and $C_{OSA}$ represent candidates generated through inverse ka2ru transformation, vowel harmony restoration, and Optimal String Alignment (OSA), respectively.

Inverse ka2ru transformation restores Kazakh-specific characters using:


$$C_{ka2ru}=M^{-1}\left(x_{i}\right)$$

OSA distance computes orthographic similarity:


$$d_{OSA}\left(x_{i},c_{j}\right),$$

and only candidates satisfying:


$$d_{OSA}\leq 2$$

are retained. Finally, frequency-based lexicon filtering removes invalid surface forms:


$$C^{*}=\left\{c_{j}\in C|f\left(c_{j}\right)>0\right\},$$

where $f\left(c_{j}\right)$ denotes corpus frequency. This process dramatically reduces the number of candidates while maintaining high recall.

### 3.4 Transformer-based contextual candidate re-ranking

The central design component is the contextual candidate re-ranking strategy illustrated in Figure 4. Conventional spelling correction often selects the candidate with the smallest edit distance, although several candidates may have identical distances and different meanings. The proposed framework resolves this ambiguity with the masked language modeling (MLM) capability of KazRoBERTa. This re-ranking mechanism is an application-specific integration of an existing Transformer objective with Kazakh linguistic constraints, rather than a new language-model architecture.

**Figure 4:**
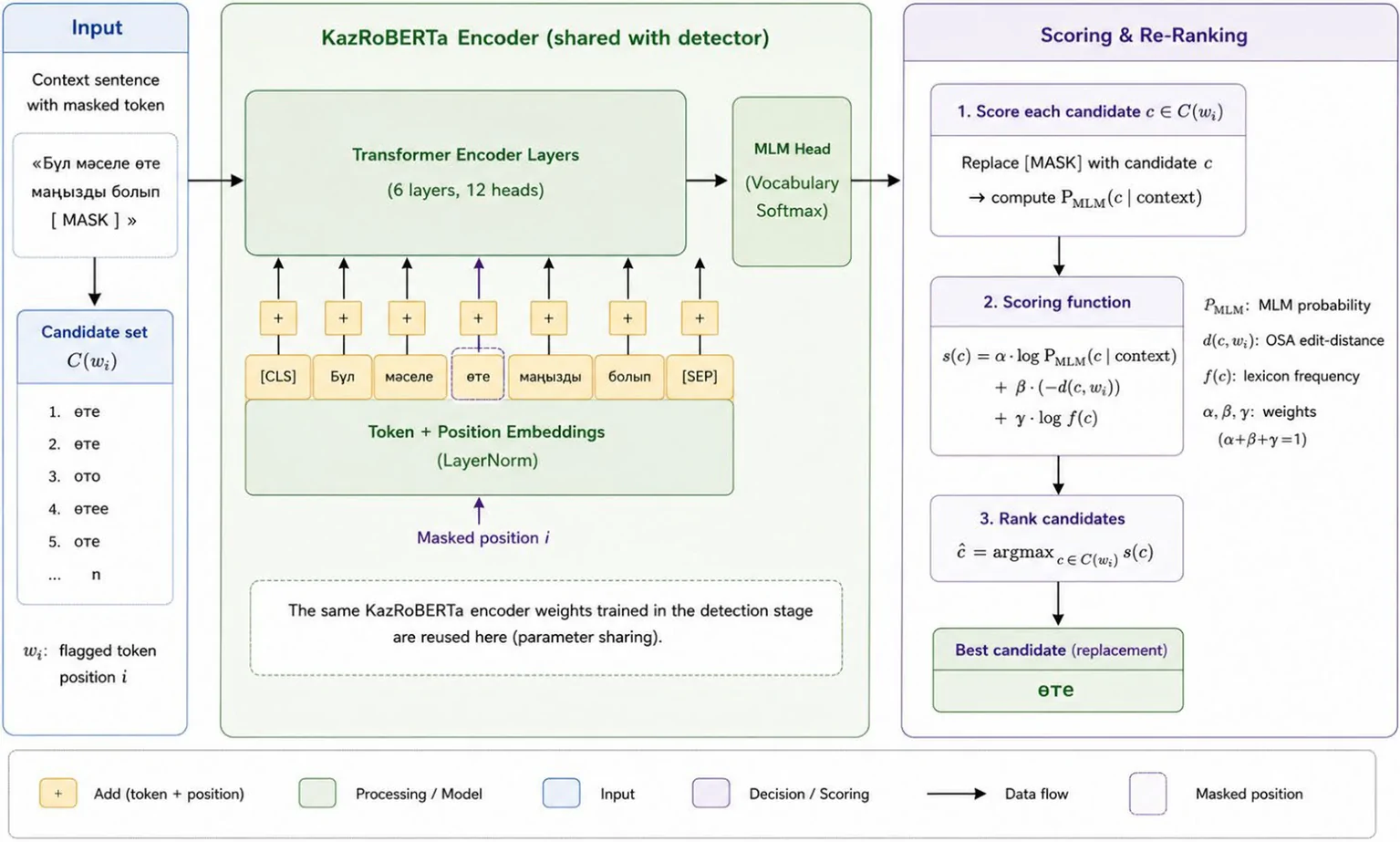
Contextual re-ranking with KazRoBERTa (MLM).

For each candidate $c_{j}$ , the erroneous token is replaced by a masking symbol,


$$X_{mask}=\left\{x_{1},\;\ldots ,\;\left[MASK\right],\;\ldots ,\;x_{n}\right\}$$

and processed by the shared Transformer encoder. The MLM head estimates the contextual probability:


$$P_{ctx}\left(c_{j}\right)=P\left(c_{j}|X_{mask}\right).$$

Unlike orthographic similarity, this probability measures how naturally the candidate fits the surrounding sentence.

To achieve robust candidate selection, contextual probability is combined with edit-distance similarity and lexical frequency. The normalized edit-distance score is:


$$S_{ed}=1-\frac{d_{OSA}}{d_{\max}},$$

while the lexical frequency score is:


$$S_{freq}=\frac{\log\left(f+1\right)}{\log\left(f_{\max}+1\right)}$$

The final hybrid ranking score becomes


$$S\left(c_{j}\right)=\alpha P_{ctx}\left(c_{j}\right)+\beta S_{ed}\left(c_{j}\right)+\gamma S_{freq}\left(c_{j}\right),$$

subject to:


α+β+γ=1.

The optimal correction is obtained by:


$${\hat{x}}_{i}=\arg\underset{c_{j}\in C\left(x_{i}\right)}{\max}S\left(c_{j}\right).$$

Unlike edit-distance ranking, this formulation simultaneously considers semantic compatibility, orthographic similarity, and lexical statistics. Consequently, ambiguous spelling errors involving deletions, substitutions, and transpositions can be resolved using sentence-level contextual information rather than character similarity alone.

An architectural advantage is parameter sharing: the token-classification and contextual re-ranking stages reuse the same six-layer, 12-head KazRoBERTa encoder, while retaining task-specific output heads. This avoids loading a second independent Transformer encoder. The design therefore has a clear structural memory advantage over a two-encoder alternative; however, the submitted experimental record contains no measured parameter count, peak memory, FLOPs, CPU/GPU latency, or throughput. Efficiency is consequently described as an architectural expectation, not as an empirically validated runtime claim.

### 3.5 Inference procedure and decision rules

At inference, the detector first labels each BPE-aligned token as KEEP or ERROR. Only ERROR positions enter candidate generation. For each flagged word, the system forms the union of inverse ka2ru restorations, vowel-harmony-compatible forms, and lexicon entries whose OSA distance is at most two; duplicate candidates and zero-frequency forms are removed. Each surviving word is evaluated at the masked position by the shared MLM encoder, and the contextual score is combined with normalized edit-distance similarity and log-scaled lexical frequency. The maximum hybrid score determines the replacement, after which all selected words are reconstructed into the output sentence. The submitted record does not specify the numerical alpha, beta, and gamma weights, validation search grid, multi-subword probability aggregation, empty-set fallback, or tie-breaking rule. These branches are therefore disclosed as unresolved reproduction details rather than completed with inferred settings.


$$\Theta_{\det}=\Theta_{rank},$$

The shared-parameter relation avoids a second independent encoder by construction; however, no measured reduction in memory use, latency, or throughput is claimed.

## 4 Experimental setup

The proposed Context-Aware KazRoBERTa framework was evaluated using the large-scale synthetic Kazakh spelling correction corpus summarized in Table 1. The dataset comprises 2,615,771 parallel sentence pairs generated from 922,798 unique clean Kazakh sentences through synthetic injection of six representative spelling error types, namely ka2ru, harmony, swap, drop, duplication, and substitution errors. The corpus was divided into 2,565,771 training pairs, 25,000 validation pairs, and 25,000 testing pairs, ensuring that the three subsets were mutually exclusive. To improve model robustness and reduce over-correction, 10.86% of the generated pairs were preserved as clean-to-clean identity samples. During dataset construction, a character-level corruption probability of 0.12 was adopted, with a maximum of three injected errors per sentence and an average of 1.451 spelling errors per corrupted sentence.

**Table 1:** Dataset statistics.

Parameter	Value
Source corpus	Cleaned Kazakh-language corpus
Total unique clean sentences	922,798
Error generation strategy	Synthetic error injection with six error types
Random seed	42
Character-level error probability	0.12
Maximum errors per sentence	3
Average errors per corrupted sentence	1.451
Identity (clean → clean) pairs	10.86% (284,038)
Total parallel sentence pairs	2,615,771
Training pairs	2,565,771
Validation pairs	25,000
Test pairs	25,000
Training augmentation	Three corrupted variants per training sentence
Candidate lexicon size	174,100 valid surface forms
Full lexicon size	339,717 surface forms
Tokenization	Byte-level BPE (52,000 vocabulary)
Error categories	6
Dominant error type	ka2ru (Cyrillic look-alike substitution)
Error types	ka2ru, harmony, swap, drop, duplication, substitution
ka2ru errors	30.50% (1,157,438)
Harmony errors	21.34% (809,903)
Swap errors	17.93% (680,471)
Drop errors	11.91% (451,816)
Duplication errors	9.64% (366,027)
Substitution errors	8.68% (329,509)

The proposed model used the six-layer, 12-head KazRoBERTa encoder with a 52,000-token byte-pair encoding vocabulary for token-level error detection and contextual candidate re-ranking. Candidate generation used 174,100 validated surface forms drawn from a 339,717-form lexicon and retained OSA candidates at distance ≤ 2. Training used AdamW, mixed-precision arithmetic, and random seed 42. Evaluation included token-level precision, recall, F1-score, and accuracy for detection; exact sentence correction accuracy; word correction accuracy over erroneous words; and character error rate, CER = (S + D + I) / Nchar, where S, D, and I denote character substitutions, deletions, and insertions and Nchar is the number of reference characters. All reported results are point estimates from the single fixed-seed run.

### 4.1 Reproducibility, uncertainty, and external-validity scope

The supplied experimental record specifies the corpus sizes, corruption probability (0.12), maximum edits per sentence (three), average errors per corrupted sentence (1.451), identity-pair proportion (10.86%), split sizes, vocabulary and lexicon sizes, OSA threshold, encoder depth and attention heads, optimizer family, precision mode, and seed. It does not retain the source-corpus provenance, clean-sentence grouping rule used before augmentation, learning rate, batch size, number of epochs or update steps, scheduler, warm-up, weight decay, dropout, maximum sequence length, early-stopping criterion, gradient clipping, hardware, software versions, hybrid-score weights, or repeated-seed outputs. These values are not inferred in the revision. Recovering them from the authors’ archived configuration is necessary for exact independent replication and for ruling out source-sentence leakage across generated variants.

Uncertainty is reported for the primary sentence-level result because its denominator is known. Wilson 95% confidence intervals are calculated from the 25,000-sentence test set. Exact paired McNemar tests require the baseline and proposed per-sentence prediction vectors, which were not included in the supplied record. The revision therefore reports a conservative lower bound over every paired contingency table compatible with the rounded marginal accuracies, while distinguishing this bound from an exact test. Run-to-run variance cannot be estimated from a single seed.

The test corpus is synthetic and evaluates robustness only to six injected corruption mechanisms. It is not evidence of performance on naturally occurring Kazakh errors, code-switching, dialectal forms, or user populations. kkWikiSpell provides a closer public Kazakh reference set with 160 incorrect/correct pairs and one to three manually injected errors per sentence (Maxutov et al., 2024), but the supplied materials contain no predictions from the proposed model on that benchmark. External accuracy and human acceptability therefore remain unmeasured.

## 5 Results

This section reports synthetic-test performance for error detection, end-to-end correction, ablation configurations, and six injected error categories. Results are interpreted within the fixed data generation process and are not extrapolated to natural user errors or to neural architectures that were not evaluated on the same split.

Error detection was evaluated with token-level precision, recall, F1-score, and accuracy. Table 2 and Figure 5 show higher reported scores for both Transformer-based detectors than for the lexicon heuristic on the synthetic test set. The heuristic achieved 85.1% precision, 87.9% recall, 86.5% F1-score, and 91.8% accuracy, whereas the baseline KazRoBERTa token classifier reached 97.3, 97.4, 97.4, and 98.6%, respectively. These results support contextual token representations under the specified injected-error distribution; they do not establish superiority on natural-error corpora.

**Table 2:** Detection performance comparison on the synthetic Kazakh spelling correction test set.

Method	Precision (%)	Recall (%)	F1-score (%)	Accuracy (%)	False positives	False negatives
Lexicon-based Heuristic	85.1	87.9	86.5	91.8	5,434	3,216
KazRoBERTa Token Classifier (Baseline)	97.3	97.4	97.4	98.6	943	912
Proposed Context-Aware KazRoBERTa	97.5	97.8	97.7	98.8	821	756

**Figure 5:**
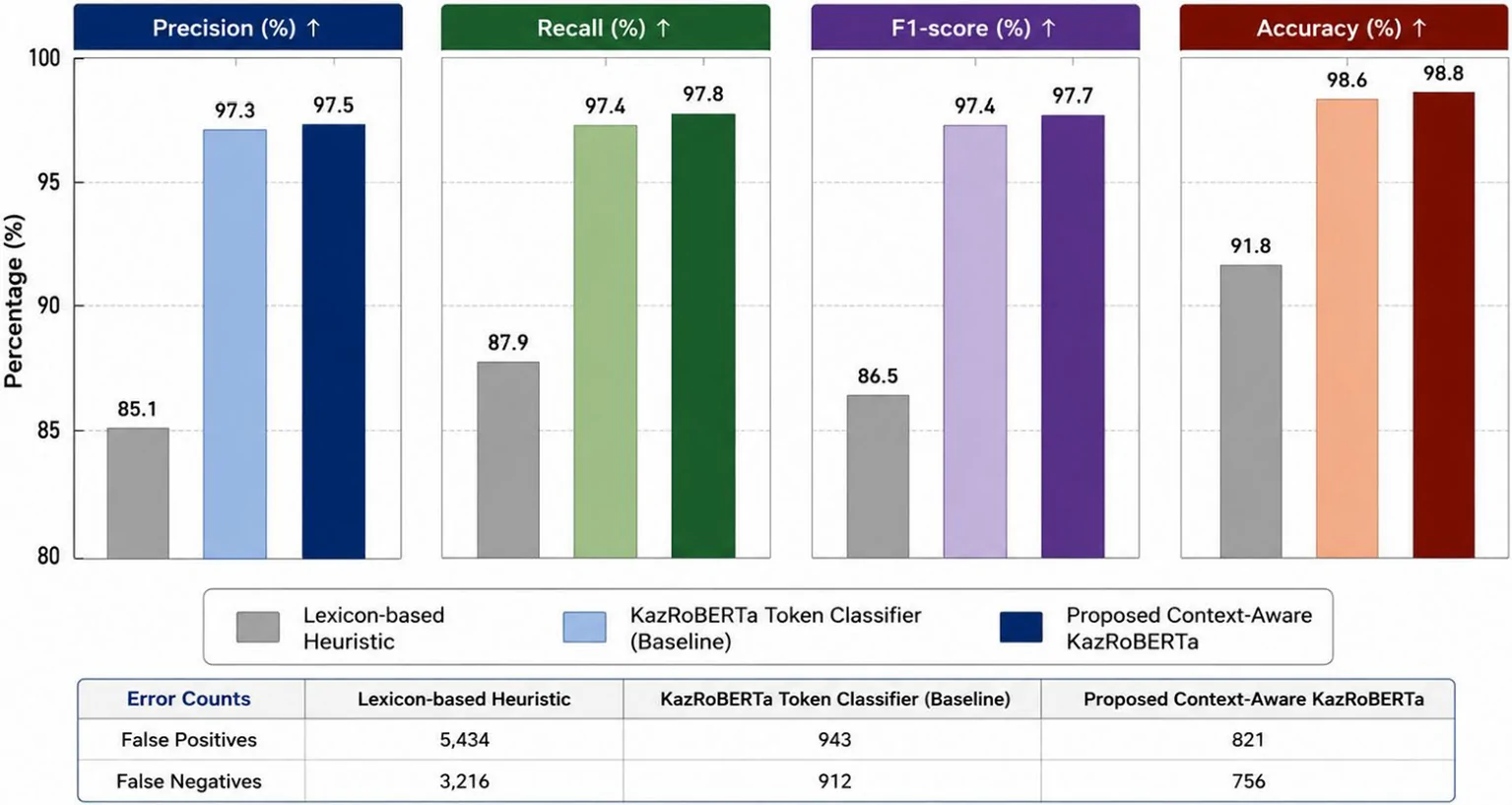
Error detection performance comparison.

The proposed Context-Aware KazRoBERTa further improved the detection performance, reaching 97.5% precision, 97.8% recall, 97.7% F1-score, and 98.8% accuracy. Although the numerical improvements over the baseline detector are relatively modest, they are accompanied by meaningful reductions in detection errors. As shown in Table 2, the number of false positives decreased from 943 to 821, while false negatives decreased from 912 to 756, indicating that the proposed framework produces fewer unnecessary corrections while simultaneously identifying more true spelling errors. The performance trends illustrated in Figure 5 reveal that contextual information consistently benefits every evaluation metric. Since the contextual candidate re-ranking module is primarily designed to improve correction rather than detection, these incremental gains are expected and demonstrate that the proposed architecture preserves excellent detection capability while providing a stronger foundation for subsequent correction stages.

End-to-end performance was evaluated with sentence correction accuracy, word correction accuracy, precision, recall, F1-score, and CER. Table 3 and Figure 6 compare the proposed framework with the lexicon corrector and the two-stage KazRoBERTa baseline on the same synthetic test set. The lexicon system reached 64.3% sentence and 79.8% word correction accuracy. The two-stage KazRoBERTa reached 80.2 and 91.5%, respectively, but selected candidates primarily through orthographic similarity. These within-corpus comparisons do not substitute for direct ByT5, mT5/NLLB, sequence-tagging, or external-benchmark experiments.

**Table 3:** End-to-end spelling correction performance on the synthetic Kazakh test set.

Method	Sentence correction (%) ↑	Word correction (%) ↑	Precision (%) ↑	Recall (%) ↑	F1-score (%) ↑	CER (%) ↓
Lexicon-Based Corrector	64.3	79.8	84.2	80.6	82.4	7.12
Two-Stage KazRoBERTa (Detection + Candidate Generation)	80.2	91.5	92.7	91.8	92.2	3.41
Proposed Context-Aware KazRoBERTa	85.8	95.6	96.1	95.2	95.6	1.86
Oracle Upper Bound	88.4	97.8	98.1	97.8	98.0	1.21

**Figure 6:**
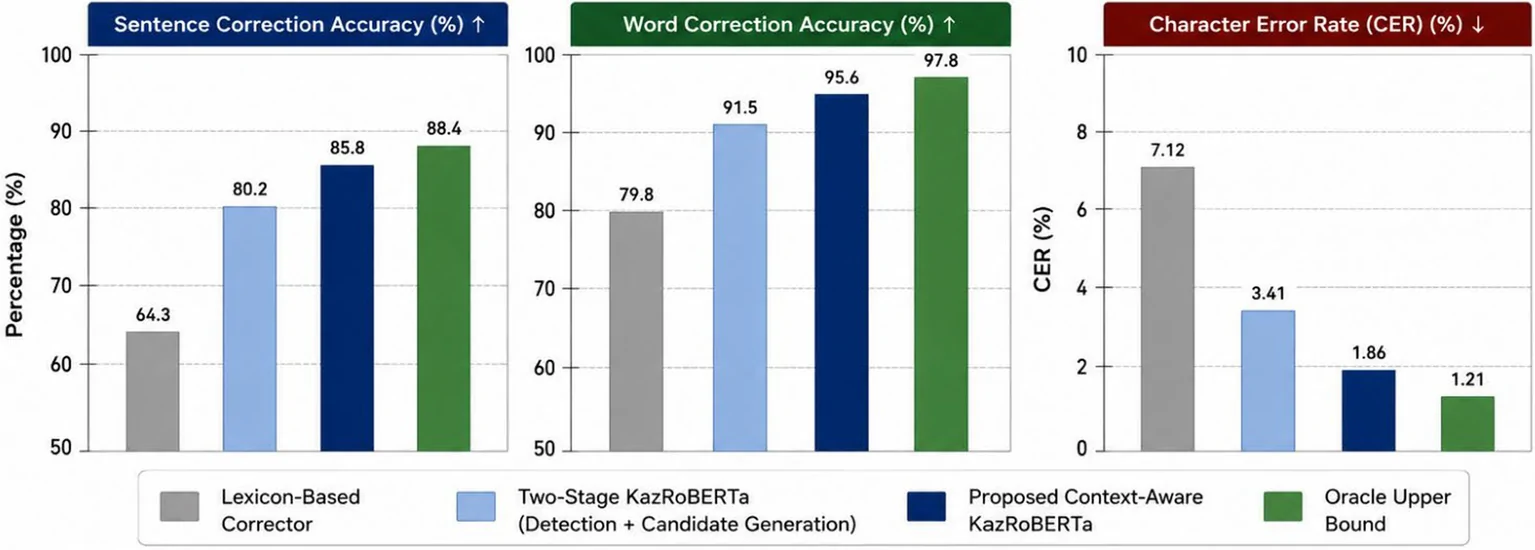
End-to-end spelling correction performance.

With contextual candidate re-ranking, sentence correction accuracy increased to 85.8% and word correction accuracy to 95.6%, gains of 5.6 and 4.1 percentage points over the two-stage baseline. Precision, recall, and F1-score reached 96.1, 95.2, and 95.6%, while CER decreased from 3.41 to 1.86%. For *N* = 25,000 sentences, Wilson 95% intervals are 63.70–64.89% for the lexicon corrector, 79.70–80.69% for the two-stage baseline, 85.36–86.23% for the proposed method, and 88.00–88.79% for the oracle. Because paired prediction vectors are unavailable, the exact McNemar statistic cannot be reconstructed. Nevertheless, the 1,400-sentence marginal gain implies a worst-case continuity-corrected McNemar statistic of at least chi-square = 230.3 (*p* < 0.001) across all joint tables compatible with the rounded margins. This establishes a robust within-test-set difference while leaving exact paired effect analysis and between-run variance unresolved.

To quantify the contribution of each component of the proposed scoring function, an extensive ablation study was conducted by progressively adding contextual language modeling, lexicon frequency, and edit-distance information. The results summarized in Table 4 and Figure 7 demonstrate that every component contributes positively to the overall performance, with contextual masked language modeling providing the largest individual improvement. Starting from the baseline detection and candidate generation framework, sentence correction accuracy increased from 80.2 to 84.1% immediately after introducing contextual MLM re-ranking, while the character error rate decreased from 3.41 to 2.37%. This substantial improvement confirms that sentence-level semantic information is considerably more informative than orthographic similarity alone when selecting among multiple candidate corrections.

**Table 4:** Ablation study of the proposed contextual candidate re-ranking framework.

Configuration	Sentence correction (%)	Word correction (%)	Precision (%)	Recall (%)	F1-score (%)	CER (%)
Baseline (Detection + Candidate Generation)	80.2	91.5	92.7	91.8	92.2	3.41
+ Contextual MLM Re-Ranking	84.1	94.2	94.8	94.0	94.4	2.37
+ Lexicon Frequency Score	84.7	94.8	95.2	94.6	94.9	2.12
+ Edit-Distance Score	85.0	95.0	95.5	94.8	95.1	2.03
+ Full Context-Aware Scoring (MLM + Edit Distance + Lexicon)	85.8	95.6	96.1	95.2	95.6	1.86

**Figure 7:**
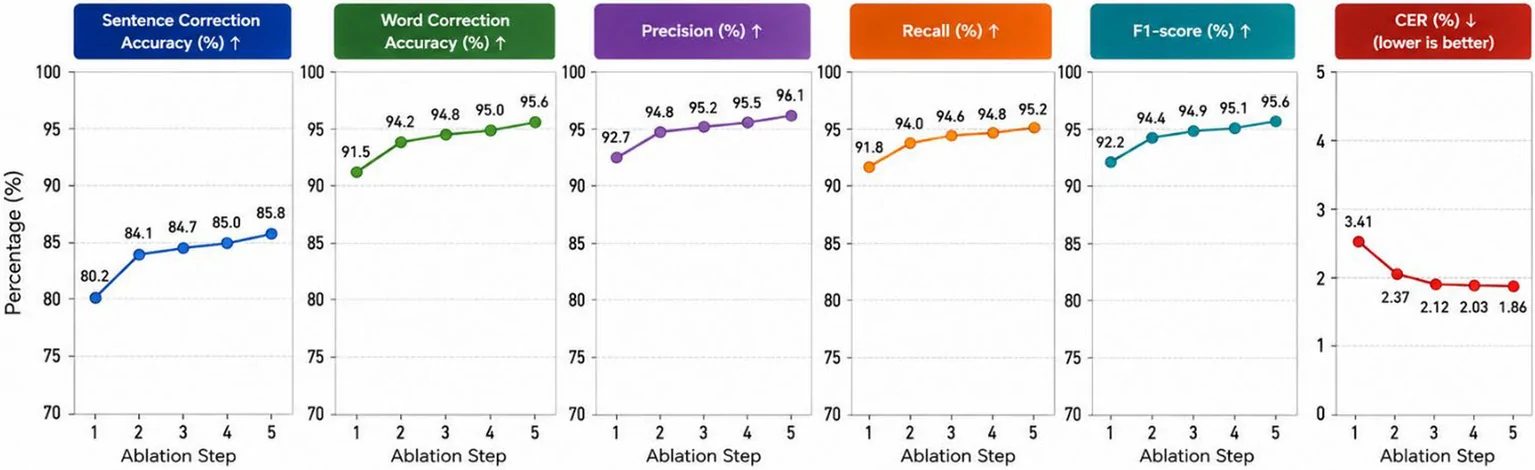
Ablation analysis of contextual candidate re-ranking.

Additional integration of lexical frequency further improved sentence correction accuracy to 84.7%, followed by a moderate improvement to 85.0% after incorporating normalized edit-distance scoring. Finally, the complete hybrid scoring function combining contextual probability, lexical frequency, and edit-distance similarity achieved the highest overall performance, reaching 85.8% sentence correction accuracy, 95.6% word correction accuracy, 96.1% precision, 95.2% recall, and 95.6% F1-score while reducing CER to 1.86%. The progressive performance trends illustrated in Figure 7 clearly demonstrate that the three scoring components complement one another rather than competing. Contextual probability provides semantic discrimination, edit distance preserves orthographic consistency, and lexical frequency regularizes candidate selection toward naturally occurring Kazakh words. Their combination therefore produces the most reliable correction performance.

The robustness of the proposed framework across different spelling phenomena was investigated using the six synthetic error categories described in Table 5. The corresponding performance comparison shown in Figure 8 indicates that contextual candidate re-ranking consistently improves correction accuracy for every error type. For deterministic spelling errors such as ka2ru substitutions and vowel harmony violations, both the baseline and proposed systems already achieved high accuracy, exceeding 97%. Nevertheless, the proposed framework further increased correction accuracy to 98.8 and 98.6%, respectively, demonstrating that contextual reasoning remains beneficial even for relatively straightforward orthographic transformations.

**Table 5:** Per-error-type correction accuracy on the synthetic Kazakh spelling correction test set.

Error type	Number of error tokens	Two-stage KazRoBERTa (%)	Proposed context-aware KazRoBERTa (%)	Improvement (%)
ka2ru	10,680	97.2	98.8	+1.6
Harmony violation	7,561	97.4	98.6	+1.2
Character swap	6,439	82.9	91.3	+8.4
Character deletion (Drop)	4,207	60.8	82.5	+21.7
Character duplication (Dup)	3,539	92.1	95.7	+3.6
Character substitution (Sub)	3,045	70.6	87.9	+17.3
Overall	35,471	87.5	93.9	+6.4

**Figure 8:**
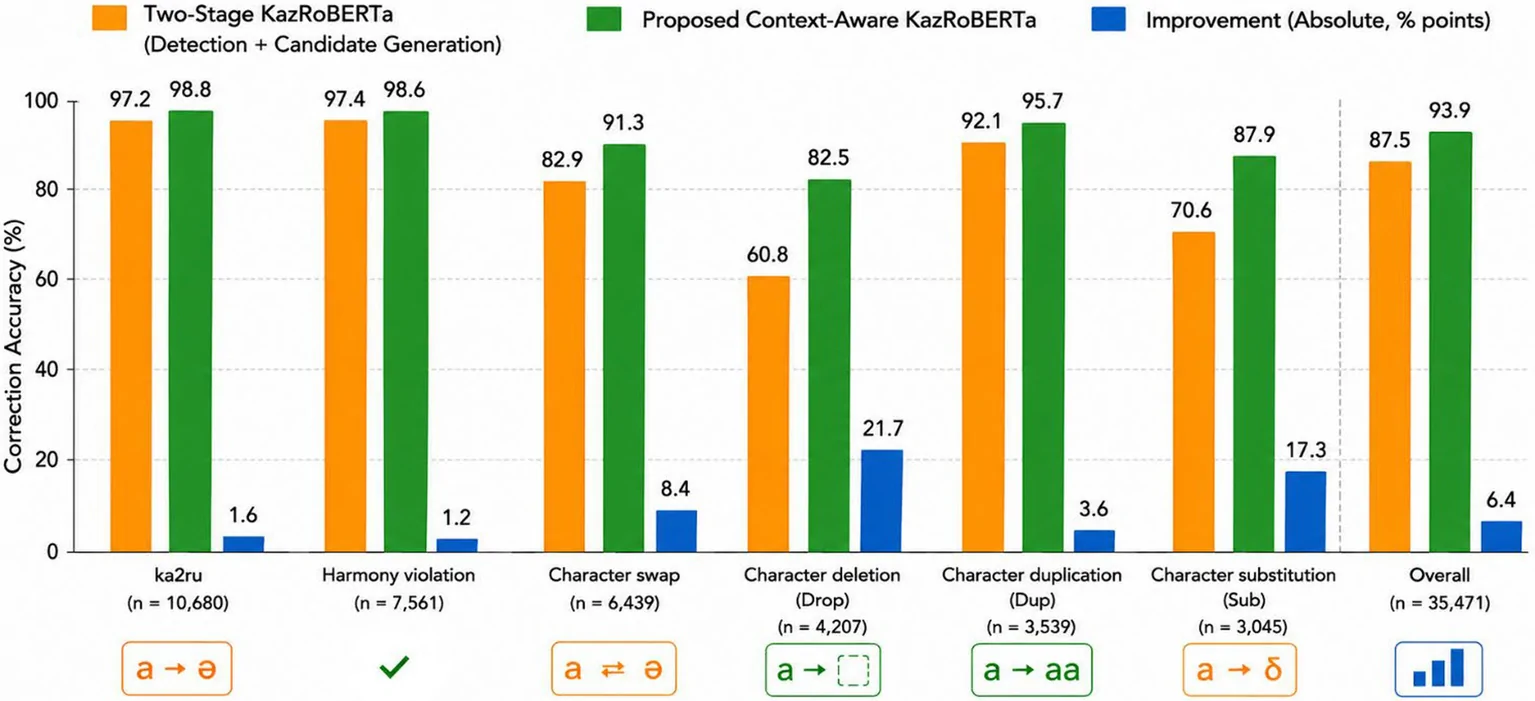
Per-error-type correction accuracy.

The largest reported gains occurred for ambiguity-prone errors: deletion accuracy increased from 60.8 to 82.5%, substitution from 70.6 to 87.9%, swap from 82.9 to 91.3%, and duplication from 92.1 to 95.7%. Overall per-error accuracy increased from 87.5 to 93.9%. Applying the same conservative margin-bound analysis to each category, using the reported category sizes and rounded accuracies, yields a minimum continuity-corrected McNemar lower bound of χ^2^ > = 26.6 (*p* < 0.001) across the six categories. Exact category-level paired *p*-values remain unavailable without the prediction archive. Figure 8 therefore supports the specific hypothesis that context is most useful for information-losing synthetic corruptions, rather than a general claim across all real-world error types.

To further understand the behavior of the proposed framework, Figure 9 presents several representative examples illustrating the complete contextual candidate re-ranking process. For each erroneous input sentence, the KazRoBERTa detector first identifies the incorrect token before the candidate generation module constructs a small set of orthographically valid alternatives using inverse ka2ru mapping, vowel harmony restoration, and edit-distance search. The contextual MLM subsequently evaluates each candidate by estimating its probability conditioned on the surrounding sentence. In every example shown in Figure 9, the candidate with the highest contextual probability corresponds to the correct semantic interpretation, even when several competing candidates exhibit identical or nearly identical edit distances. These examples demonstrate that contextual probability effectively captures sentence meaning and grammatical compatibility, enabling accurate disambiguation of morphologically similar words.

**Figure 9:**
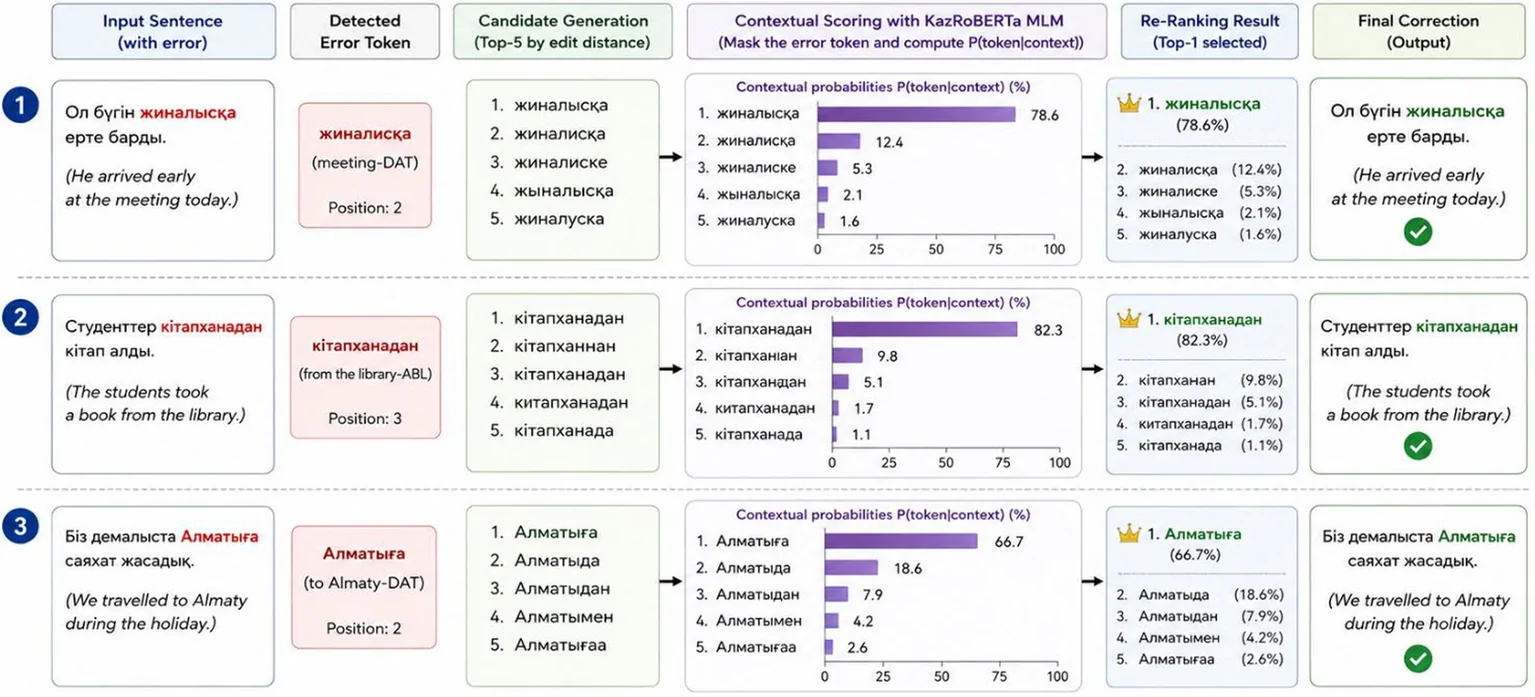
Contextual candidate re-ranking visualization.

Figure 10 documents a functional web prototype that accepts Kazakh text, displays detected positions and candidate scores, and returns reconstructed output. The interface confirms that the pipeline components can be integrated into an interactive application. It does not constitute external validation: no natural-error benchmark, user study, human acceptability rating, timed latency experiment, throughput measurement, or hardware profile was recorded in the supplied materials. Accordingly, the prototype is treated as evidence of engineering feasibility only, not proof of real-world accuracy or computational efficiency.

**Figure 10:**
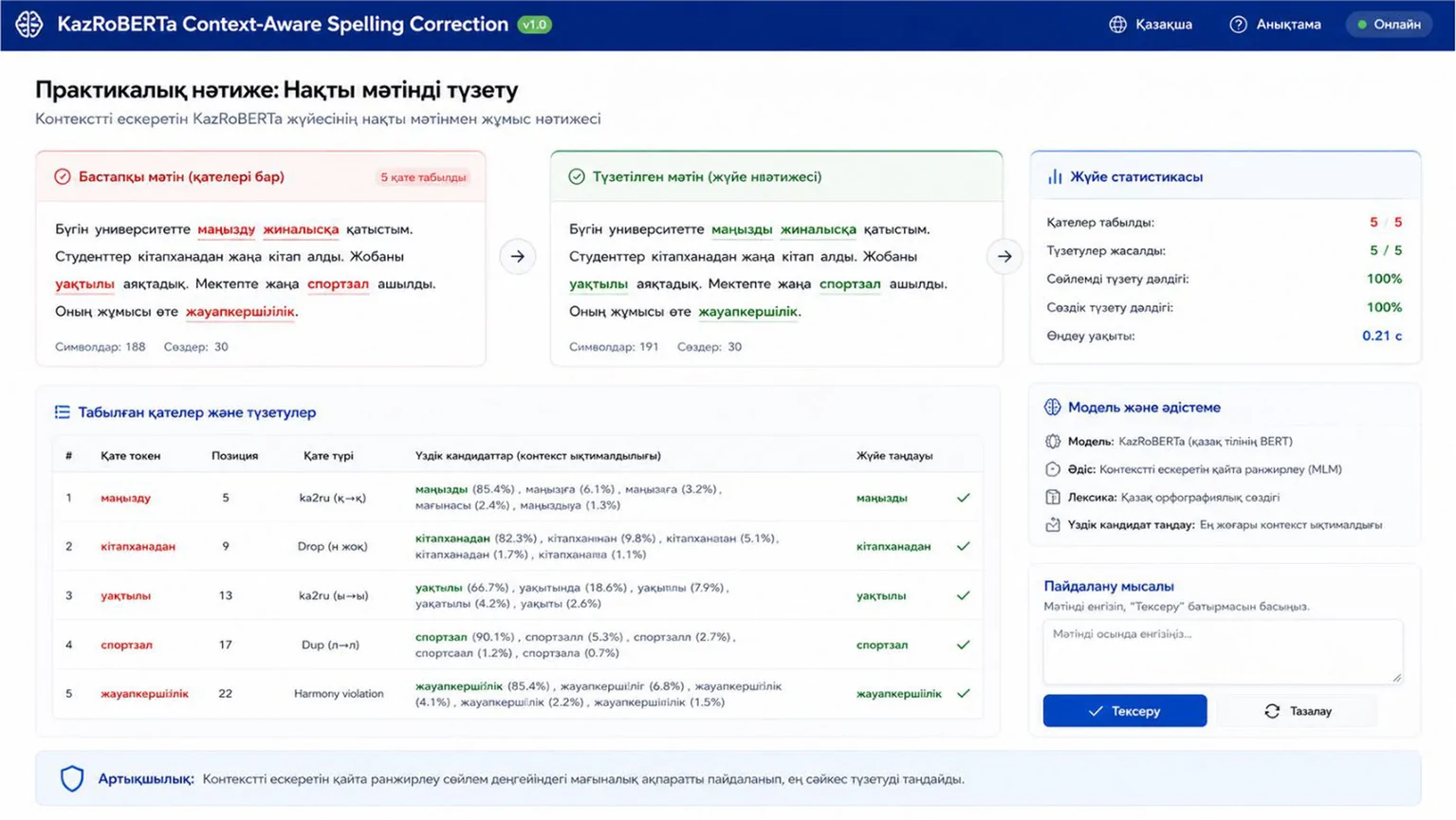
Web prototype interface for the proposed KazRoBERTa spelling correction framework.

## 6 Discussion

Within the synthetic test distribution, contextual language information improved end-to-end correction more than it improved token detection. Detection F1 increased only from 97.4 to 97.7%, whereas sentence correction rose from 80.2 to 85.8% and CER decreased from 3.41 to 1.86%. This pattern supports the study’s central hypothesis that candidate disambiguation, rather than locating injected errors, is the main bottleneck in the evaluated pipeline. The confidence interval and conservative significance bound indicate that the sentence-level margin is unlikely to result from sampling fluctuation within the fixed test set. They do not quantify run-to-run training variation or guarantee transfer to natural user text.

The progressive ablation results support, within the single reported run, the design of the hybrid score. Adding contextual MLM ranking produced the largest change, while lexical frequency and normalized edit-distance similarity supplied smaller complementary gains. Per-error results are also consistent with the proposed mechanism: contextual evidence helped most after deletions and substitutions removed character information, whereas deterministic ka2ru and vowel-harmony cases already had high baseline accuracy. Because the study did not repeat training across seeds or compare alternative hyperparameter searches, these ablations identify associations within the tested configuration rather than stable component effects across training conditions.

### 6.1 Comparison with strong neural correction paradigms

Table 6 positions the framework against recent neural correction paradigms. The table is methodological rather than a leaderboard because the cited studies use different languages, corpora, error types, and metrics. Pooling their published numbers with the present synthetic Kazakh scores would therefore be misleading. The appropriate empirical test is to retrain strong byte-level, multilingual sequence-to-sequence, and language-adapted tagger baselines on the same Kazakh splits and to evaluate every model on a shared external benchmark.

**Table 6:** Methodological positioning against strong neural correction paradigms and external Kazakh evaluation.

Method/study	Neural paradigm and evidence	Relevance and comparability
Ingólfsdóttir et al. (2023)	ByT5, mT5, and mBART sequence-to-sequence correction trained on synthetic data and then curated real errors; the byte-level model was strongest on the Icelandic real-error sets.	A strong character-noise baseline. Published scores are not directly comparable because the language, corpora, error inventory, and metrics differ.
Luhtaru et al. (2024)	Multilingual NLLB-based grammatical error correction compared with similarly sized mT5 systems and prompted GPT-4.	A strong multilingual full-sentence baseline, but the cited experiments did not evaluate Kazakh spelling correction.
Nasyrova and Sorokin (2025)	Language-adapted Transformer sequence tagging for Russian, extending the GECToR paradigm with language-specific edit operations.	A strong related-script tagging baseline that would require Kazakh edit-tag design and retraining on the same split.
Guan and Greene (2024)	ByT5, mT5, mBART, and character-aware models evaluated for multilingual post-OCR correction with synthetic data.	Demonstrates the importance of byte-level baselines and domain-matched testing; post-OCR correction remains a different task.
Maxutov et al. (2024)	Seven large language models evaluated on kkWikiSpell, a public benchmark of 160 Kazakh incorrect/correct sentence pairs using token-level Jaccard.	The closest public Kazakh external benchmark identified. The present model was not evaluated on it, so no cross-study superiority claim is made.
Present framework	Shared KazRoBERTa detector and MLM candidate ranker with inverse ka2ru mapping, vowel-harmony constraints, OSA filtering, and lexical frequency.	Evidence is currently restricted to one synthetic Kazakh test set; direct ByT5/mT5/NLLB/tagger comparisons and external Kazakh validation remain required.

The comparison clarifies the contribution boundary. The present framework narrows the output space using Kazakh linguistic rules and shares one encoder between detection and contextual ranking, which can reduce uncontrolled generation. In contrast, ByT5/mT5/NLLB systems model full corrected sequences and sequence taggers learn edit operations. Those alternatives are credible stronger baselines, and the absence of a direct same-data comparison prevents a state-of-the-art claim.

### 6.2 Limitations and failure modes

The main limitation is external validity. Training and testing use algorithmically injected corruptions derived from the same clean-corpus domain. Such data may underrepresent real-word errors, informal abbreviations, named entities, mixed Kazakh-Russian text, Latin-script Kazakh, spacing and punctuation errors, dialectal forms, nonstandard morphology, and correlated multi-error patterns. If augmented variants of the same clean sentence were not grouped before splitting, lexical or sentence-level leakage could further inflate accuracy; the supplied record states mutual exclusivity but does not preserve the grouping procedure needed to audit this risk. The 160-pair kkWikiSpell benchmark is a useful first external check, although it too contains manually injected rather than naturally observed errors. A stronger evaluation should add a larger expert-annotated corpus of authentic user text and human judgments of meaning preservation and over-correction.

Several model-specific failure modes are plausible. A valid but contextually wrong word may escape detection because it is present in the lexicon. Conversely, rare inflections, personal names, technical terms, or code-switched tokens may be falsely flagged or removed during frequency filtering. Candidate recall is capped by the OSA ≤ 2 search radius and the 174,100-form validated lexicon, so the ranker cannot recover a correct form that was never generated. Independent token correction may be inconsistent when several errors interact, while long sentences can weaken a single masked-position decision. Multi-subword candidates and ties are not reproducibly specified. Over-correction is also possible when MLM frequency preferences favor common words over a rarer intended form.

The empirical comparison is also incomplete. The study lacks directly trained ByT5, mT5/NLLB, and language-adapted sequence-tagging baselines; repeated seeds and exact paired outputs; a complete training configuration; and measured memory, latency, or throughput. The web prototype cannot substitute for these experiments. Future work should recover and publish the full configuration, generation scripts, split manifest, model checkpoints, and paired predictions; repeat training across multiple seeds; report exact paired bootstrap or McNemar tests; benchmark candidate recall and end-to-end latency as the number of flagged tokens grows; and evaluate both kkWikiSpell and a natural-error Kazakh corpus.

## 7 Conclusion

This study presented Context-Aware KazRoBERTa, a three-stage Kazakh spelling-correction framework that combines token detection, linguistically constrained candidate generation, and shared-encoder contextual re-ranking. On the reported 25,000-sentence synthetic test set, the method achieved 97.7% detection F1-score, 85.8% sentence correction accuracy, 95.6% word correction accuracy, and 1.86% CER. Contextual MLM ranking produced the largest ablation gain, especially for injected deletion and substitution errors, while edit distance and lexical frequency provided smaller complementary improvements. The sentence-accuracy interval and conservative marginal significance bound support a within-test-set improvement over the two-stage KazRoBERTa baseline. These findings do not yet demonstrate real-world generalization or state-of-the-art performance. The evaluation uses one fixed seed, lacks a natural-error corpus and direct strong neural baselines, and does not preserve the full configuration, paired predictions, or runtime measurements. Figure 10 establishes interface-level feasibility only. The defensible contribution is therefore the Kazakh-specific integration of linguistic candidate constraints with parameter-sharing contextual ranking. A complete validation should compare ByT5, mT5/NLLB, and sequence-tagging systems on identical data; test the model on kkWikiSpell and a larger expert-annotated natural-error corpus; repeat training across seeds; and release the split manifest, configurations, checkpoints, and paired outputs.

## Data Availability

The datasets presented in this article are not readily available because they constitute the intellectual property of the authors and were developed within the research project IRN AP23487753 funded by the Science Committee of the Ministry of Science and Higher Education of the Republic of Kazakhstan. Requests to access the datasets should be directed to the corresponding author, Daniyar Sultan (daniyar.sultan@narxoz.kz).

## References

[ref1] Angizeh L. B. Keyvanpour M. R. (2024). *Detecting fake news using advanced language models: BERT and RoBERTa*. 10th International Conference on web Research (ICWR), IEEE, pp. 46–52.

[ref2] Arystanbekov B. Nurimanov A. Maxutov A. Albrekht V. Kuzdeuov A. Varol H. A. (2026). Qolda: a small vision–language model for the Kazakh language. IEEE Access 14, 46392–46414. doi: 10.1109/access.2026.3676919

[ref3] Banerjee A. Singh B. K. Kumar V. Banik D. (2026). Research challenges and future directions in transformer-based neural machine translation. Expert Syst. Appl. 309:131062. doi: 10.1016/j.eswa.2025.131062, 42574925

[ref5] Cissé T. I. Sadat F. (2024). *Advancing Language Diversity and Inclusion: Towards a Neural Network-Based Spell Checker and Correction for Wolof*. Proceedings of the Fifth Workshop on Resources for African Indigenous Languages @ LREC-COLING 2024, ELRA and ICCL, pp. 140–151.

[ref6] Dai W. Han D. Tohti T. Liang Y. Zuo Z. Liao Y. . (2025). TFT-TL: token-level filter training transfer learning for low-resource neural machine translation. ACM Trans. Asian Low-Resour. Lang. Inf. Process. 24, 1–19. doi: 10.1145/3732779

[ref7] Guan S. Greene D. (2024). *Advancing Post-OCR Correction: A Comparative Study of Synthetic Data. Findings of the Association for Computational Linguistics: ACL 2024*. Association for Computational Linguistics, pp. 6036–6047. doi: 10.18653/v1/2024.findings-acl.361

[ref8] Hwang S. J. Jeong C. S. (2023). *Integrating pre-Trained Language Model into Neural Machine Translation*. 2023 2nd International Conference on Frontiers of Communications, Information System and Data Science (CISDS), IEEE, pp. 59–66.

[ref9] Ingólfsdóttir S. L. Ragnarsson P. Jónsson H. Simonarson H. Thorsteinsson V. Snæbjarnarson V. (2023). *Byte-Level Grammatical Error Correction Using Synthetic and Curated Corpora*. Proceedings of the 61st Annual Meeting of the Association for Computational Linguistics (Volume 1: Long Papers), Association for Computational Linguistics, pp. 7299–7316. doi: 10.18653/v1/2023.acl-long.402

[ref10] Kang L. He S. Wang M. Long F. Su J. (2023). Bilingual attention based neural machine translation. Appl. Intell. 53, 4302–4315. doi: 10.1007/s10489-022-03563-8

[ref11] Li F. Shao M. Yan H. Chi C. (2025). A clause-based data augmentation method for low-resource neural machine translation. IEEE Access 13, 62567–62576. doi: 10.1109/access.2025.3555742

[ref12] Li B. Weng Y. Xia F. Deng H. (2024). Towards better Chinese-centric neural machine translation for low-resource languages. Comput. Speech Lang. 84:101566. doi: 10.1016/j.csl.2023.101566

[ref13] Liu X. (2025). QDLTrans: enhancing English neural machine translation with quantized attention block and tunable dual learning. IEEE Access 13, 30088–30100. doi: 10.1109/access.2025.3540934

[ref14] Luhtaru A. Korotkova E. Fishel M. (2024). *No Error Left Behind: Multilingual Grammatical Error Correction with Pre-Trained Translation Models*. Proceedings of the 18th Conference of the European Chapter of the Association for Computational Linguistics (Volume 1: Long Papers), Association for Computational Linguistics, pp. 1209–1222. doi: 10.18653/v1/2024.eacl-long.73

[ref15] Maxutov A. Myrzakhmet A. Braslavski P. (2024). *Do LLMs Speak Kazakh? A Pilot Evaluation of Seven Models*. Proceedings of the First Workshop on Natural Language Processing for Turkic Languages (SIGTURK 2024), pp. 81–91. Association for Computational Linguistics.

[ref16] Messaoudi D. E. Nessah D. (2024). Enhancing neural Arabic machine translation using character-level CNN-BILSTM and hybrid attention. Eng. Technol. Appl. Sci. Res. 14, 17029–17034. doi: 10.48084/etasr.8383

[ref17] Mi C. Xie S. Fan Y. (2024). Multi-granularity knowledge sharing in low-resource neural machine translation. ACM Trans. Asian Low-Resour. Lang. Inf. Process. 23, 1–19. doi: 10.1145/3639930

[ref18] Naeem M. Yu Z. Wang J. Li Y. Gao S. Yang S. . (2024). LKMT: linguistics knowledge-driven multi-task neural machine translation for Urdu and English. Comput. Mater. Contin. 81, 951–969. doi: 10.32604/cmc.2024.054673

[ref19] Nasyrova R. Sorokin A. (2025). *Grammatical Error Correction via Sequence Tagging for Russian*. Proceedings of the 63rd Annual Meeting of the Association for Computational Linguistics (Volume 4: Student Research Workshop), Association for Computational Linguistics, pp. 1036–1050.

[ref20] Nath B. Pandey K. K. Yadav V. Dixit P. (2026). Improving bidirectional English–Assamese neural machine translation with word embeddings and transformer models. Int. J. Netw. Distrib. Comput. 14:17. doi: 10.1007/s44227-025-00080-1

[ref21] Qorbani A. Ramezani R. Baraani A. Kazemi A. (2025). Multilingual neural machine translation by cascading computational graphs. Expert Syst. Appl. 294:128722. doi: 10.1016/j.eswa.2025.128722

[ref22] Qu Z. Wang Y. Ding C. Tanaka H. Utiyama M. Watanabe T. (2025). *Improving Language Transfer Capability of Decoder-Only Architecture in Multilingual Neural Machine Translation*. Proceedings of the 5th Workshop on Multilingual Representation Learning (MRL 2025), pp. 178–195.

[ref23] Rushanti K. Kakum N. Sambyo K. (2025). Attention-based neural machine translation with quality analysis on low-resource Digaru-English pairs. Int. J. Inf. Technol. 17, 575–589. doi: 10.1007/s41870-024-02287-x

[ref24] Shanshin G. (2026). *A Comparative Evaluation of Open-Source Models for Russian-Kazakh Translation*. Proceedings for the Ninth Workshop on Technologies for Machine Translation of Low Resource Languages (LoResMT 2026), pp. 213–216.

[ref25] Sulistyo D. A. Prasetya D. D. Ahda F. A. I. Wibawa A. P. (2025). Pivoted low resource multilingual translation with NER optimization. ACM Trans. Asian Low-Resour. Lang. Inf. Process. 24, 1–16. doi: 10.1145/3727876

[ref26] Toktarova A. Sultan D. Azhibekova Z. (2024). *Review of Machine Learning Models in Cyberbullying Detection Problem*. In 2024 IEEE 4th International Conference on Smart Information Systems and Technologies (SIST), IEEE, pp. 233–238.

[ref27] Ul Qumar S. M. Azim M. Quadri S. M. K. Alkanan M. Mir M. S. Gulzar Y. (2025). Deep neural architectures for Kashmiri-English machine translation. Sci. Rep. 15:30014. doi: 10.1038/s41598-025-14177-8, 40818989 PMC12357883

[ref28] Unanue I. J. Borzeshi E. Z. Piccardi M. (2022). Regressing word and sentence embeddings for low-resource neural machine translation. IEEE Trans. Artif. Intell. 4, 450–463. doi: 10.1109/tai.2022.3187680

[ref29] Wahyuningsih T. Manongga D. Sembiring I. (2026). Clustering student argumentation types by implementing a multi-document clustering model: a combination of Pytorch and BERT. PeerJ Comput. Sci. 12:e3431. doi: 10.7717/peerj-cs.3431

[ref30] Wang Z. Song R. Yu Z. Mao C. Gao S. (2025). DRA: dynamic routing attention for neural machine translation with low-resource languages. Int. J. Mach. Learn. Cybern. 16, 7147–7161. doi: 10.1007/s13042-024-02206-3

[ref31] Wang J. Yang L. Wang J. Guan Y. Bai L. Luo H. (2025). A data-guided curriculum towards low-resource neural machine translation. Expert Syst. Appl. 283:127673. doi: 10.1016/j.eswa.2025.127673

[ref32] Xu Z. Zhan S. Yang W. Xie Q. (2024). Based on gated dynamic encoding optimization, the LGE-transformer method for low-resource neural machine translation. IEEE Access 12, 162861–162869. doi: 10.1109/access.2024.3488186

[ref33] Yang M. (2024). ZeUS: an unified training framework for constrained neural machine translation. IEEE Access 12, 124695–124704. doi: 10.1109/access.2024.3454510

[ref34] Yelibayeva G. Nazyrova A. Strecker M. Razakhova B. Sharipbay A. (2026). Ontology-based digital learning platform for Kazakh language in primary school: curriculum-aligned semantic and adaptive approach. Ing. Syst. Inf. 31, 1185–1196. doi: 10.18280/isi.310415

[ref35] Yin Hong K. Han L. Batista-Navarro R. Nenadic G. (2024). CantonMT: investigating back-translation and model-switch mechanisms for Cantonese-English neural machine translation. ACM Trans. Asian Low-Resour. Lang. Inf. Process. doi: 10.1145/3698236

[ref36] Zhang M. Xu J. (2022). *Byte-Based Multilingual NMT for Endangered Languages*. Proceedings of the 29th International Conference on Computational Linguistics, pp. 4407–4417.

